# Home Fae Home: A case study in co‐designing trauma‐informed community spaces with young people in Dundee, Scotland

**DOI:** 10.1111/camh.12765

**Published:** 2025-04-18

**Authors:** Charis Robertson, Gary Kennedy, Linsey McIntosh, Anne McKechnie

**Affiliations:** ^1^ Hot Chocolate Trust Dundee UK; ^2^ University of Dundee Dundee UK; ^3^ KennedyTwaddle London UK; ^4^ RIBA Chartered Architects Dundee UK; ^5^ Independent Designer Dundee UK; ^6^ Independent Consultant Forensic and Clinical Psychologist Glasgow UK

**Keywords:** Trauma, adolescence, creativity, community programmes, environment

## Abstract

**Background:**

*Home Fae Home* was an action research project, working with young people in Dundee, Scotland, to redesign the interior environment of a community‐based youth work facility, through the lens of trauma‐informed practice. Multi‐disciplinary in nature, the project integrated the fields of psychology, architecture and spatial design with youth and community work.

**Methods:**

Over 150 young people were engaged over 4 years in the co‐design process through a range of creative workshops. As part of these workshops, a multi‐methods approach to data collection was used, with semi‐structured interviews and focus groups, as well as more creative and informal engagements.

**Results:**

Spatially, the project has provided six new/additional diverse and adaptable youth work spaces, each thoughtfully designed by young people, to help them feel safe, process complex emotions, support recovery, avoid re‐traumatization and reduce stress levels. Through the process, important new knowledge was also generated by the young people, highlighting the importance of expression and culture for adolescents and their need to have choice and ownership of their space.

**Conclusions:**

The project clearly demonstrated that young people should be trusted as experts of their own experience of trauma and recovery and evidenced the crucial need for professionals who work with young people to deliberately redress power imbalances in order to facilitate this.

**Key Practitioner Message**

**What is known?**

- Very little has been published about the impact of the physical environment on the potential recovery from trauma.
- Literature that does exist tends to be from clinical contexts, adult perspectives, and draws on tertiary educated middle‐class cultural assumptions.

**What is new?**

- This study has pioneered an innovative action research process, which both seeks to generate new knowledge and tangibly solve a real‐life problem.
- Over 150 young people became co‐designers in a real‐life capital project, alongside designers, psychologists, and youth workers, to recreate their community space through the lens of trauma‐informed practice.
- As a result, this study has generated important new knowledge specifically with and from young people about their experiences, ideas, and needs for a real‐life physical environment to help with their recovery from trauma.

**What is significant for clinical practice?**

- The Home Fae Home has profoundly evidenced the need for professionals and services to: proactively rebalance power imbalances with young people; to set aside their own cultural norms and assumptions; and to trust young people to be the experts of their own experiences.

## Background

This place works because it feels like a teenager's bedroom.– Young person, Hot Chocolate Trust

### Hot Chocolate Trust

Hot Chocolate Trust [HCT] is a grassroots youth work organisation in Dundee, Scotland. Operating since 2001, the organisation has a strong track record in building community with young people who hang out in the city centre and who are aged 12–25. These young people are bright, articulate, creative, and also frequently face multiple life complexities and adversities. Often marginalised from their homes, schools and neighbourhoods, they regularly express that they consider HCT to be a key place of safety. They feel a deep sense of belonging there, where they can be themselves and where they can express themselves. It is their ‘home fae home’ (in Scots parlance) (Hot Chocolate Trust, 2024).

HCT's approach is underpinned by two main theoretical frameworks: (1) Youth work (participation must be voluntary; starting where young people are at; and prioritising mutual, trusting relationships based on learning together; Batsleer & Davies, 2010; Youthlink Scotland, 2024); and (2) community development (working *with* not *for* people; valuing community members as the experts of their experience; facilitating collective activism; Butcher, Banks, Henderson, & Robertson, 2007; Freire, 1993).

These underlying principles have helped establish a strong, dynamic, and trusting community between young people and youth workers, where young people feel a great sense of ownership of and pride in their space. Trust is built through creating shared experiences between young people and youth workers, and, crucially, from sharing the power and responsibility for the community.

With that trust comes a third form of sharing: the sharing of life circumstances. Many of the young people who attend HCT have, often because of trauma and adversity, had services forced upon them with little choice or control. They are commonly mistrustful of statutory services and the associated formal environments. Their experience of HCT is fundamentally different: involvement is entirely voluntary, and initial engagement normally happens because their peers bring them. Because of this, young people tend to enter assuming that HCT is a safe space, that the adults who work there can be trusted, and whereby they feel able to disclose their experiences of adversity and trauma.

### Trauma‐informed policy context

In 2017, the Scottish Government made clear its intentions to make trauma‐informed practice [TIP] a policy priority by including it in its national strategy paper (Scottish Government, 2017) and by supporting the National Health Service Education Scotland [NES] to implement a Trauma Training Framework (2015). This latter document outlined core skills and knowledge required by all levels of the workforce within Scotland to ensure that the needs of adults, young people, and children affected by trauma are met. This has been developed further with the publication of the Justice Framework and the Roadmap for Creating Trauma‐informed and Response Change Guidance for Organisations, both launched in 2023.

These developments are not exclusive to Scotland and mirror similar initiatives worldwide, including the work of the pioneering US‐based Substance Abuse and Mental Health Services Administration (SAMHSA). Their 2014 publication ‘Concept of Trauma and Guidance for a Trauma‐Informed Approach’ defines a trauma‐informed organisation as one which “realizes the widespread impact of trauma and understands potential paths for recovery; recognizes the signs and symptoms of trauma in clients, families, staff, and others involved with the system; and responds by fully integrating knowledge about trauma into policies, procedures, and practices, and seeks to actively resist re‐traumatization.” (SAMHSA, 2024 p. 9).

The key trauma‐informed principles articulated in that same document by SAMHSA have been adopted by many organisations across the world, and indeed by NES in a Scottish context. The principles are Choice, Collaboration, and Empowerment in order to build a sense of Trust and feelings of Safety (2024, p. 10). All of which are absent when a child or adult is being abused.

Trauma‐informed approaches (TIA) recognize the high prevalence of trauma and realize its impact on people's everyday functioning. There is a desire from organizations using TIA to create safe environments for both clients and staff and avoid replicating the conditions under which the abuse took place, either in adulthood or childhood. The approach recognizes the potential inability to feel safe with and trust others, particularly those with power and authority. In acknowledging the specific impact of interpersonal abuse, such organizations aim to build trust and safety through processes of collaboration, choice, and empowerment, thus improving engagement, avoiding re‐traumatization, and supporting recovery through the development of safe and healing relationships.

### Policy into practice

HCT had already been thinking deeply about this area for a number of years, and the emergence of national policies helped catalyse a strategic organisational TIP development plan.

One early action was to establish a partnership with a Consultant Clinical Psychologist who could help the senior management and youth workers to recognize the ways that the impact of trauma can occur and present across the community, and work to support young people's safety, stabilization, and recovery (Herman, 2015). For over a decade, this Consultant Clinical Psychologist provided advice, specialist training, coaching, policy development, and clinical supervision to help build HCT as a trauma‐informed organization, in line with the Trauma Training Framework (NES, 2017).

This partnership has enabled the team at HCT to better understand the well‐established principles of trauma‐informed practice: the need for a safe environment where trust is earned through collaboration, empowerment, and offering choice wherever possible. And, crucially, it has also helped the youth workers see that much of their work was already trauma‐informed and therapeutic (with a small ‘t’).

### Home FAE Home

One aspect of HCT's context which was less conducive to TIP, however, was its physical environment.

Based in a voluminous historical church, HCT was not short of space, but it had outgrown the building's existing configuration. The young people frequently expressed their hopes and needs for alternative spaces to help them regulate their anxiety, reduce their stress levels, and express their emotions. In 2018, HCT successfully secured funding to undertake a sizeable capital development project, fittingly named: Home Fae Home.

The aim of Home Fae Home was to redesign the physical space through the lens of TIP, with young people as co‐designers.

When Home Fae Home began, almost no available literature or research could be found regarding the role of physical environments in the recovery from trauma. The little that did exist tended to be from more clinical contexts, from adult perspectives, and inevitably drew on tertiary educated middle‐class cultural assumptions (Connellan et al., 2013; Jackson, 2018). The community of young people at Hot Chocolate is predominantly working class; LGBTQ+; and from ‘alternative’ subcultures including skaters, emos and goths (their language).

From the outset, it became clear that the project must be designed as action research to ensure that the young people were positioned and valued as the experts of their own experience. A local design duo who specialize in community engagement processes within the world of spatial/architectural design was appointed as partners for the project, along with the existing Consultant Clinical Psychologist. This is where trauma‐informed approaches began to enter the field of design and the built environment.

## Methods

In action research, the process both seeks to generate new knowledge and offer tangible solutions to an issue (McNiff, 2017). By adopting this research paradigm, the young people (with HCT team) became co‐designers who offered tangible solutions to the problematic nature of HCT's interior environment, as well as co‐researchers who generated new knowledge.

Through 4 years of the Home Fae Home project, over 150 young people were involved via creative workshops, drop‐in sessions, focus groups, and 1–1 conversations. The research, whilst firmly rooted within a youth work practice context, ensured that the project plan, research design, informed consent processes, data collection, storage and analysis were all in line with the Community Learning & Development Standards Council of Scotland's Code of Ethics (2017) as well as HCT's own rigorous consent and data protection policies and procedures.

### Co‐design

A core value of youth work and co‐design are shared. The heart of co‐design, according to McKercher, is to design ‘with, not for, people’ (McKercher, 2020, p. 14). It requires a shift in power from a ‘normal’ design process where designers are regarded as the experts, to a more radical stance that values all involved (in this case, the young people, HCT staff and volunteers, Consultant Clinical Psychologist, and designers) as equal members of the co‐design team. It was vitally important to the designers to ensure that the young people were involved at every stage of the design decision making process and not just as a tokenistic gesture.

To maximise the breadth and depth of the young people's involvement and decision making, the designers took time before the project began to better understand trauma and also to build relationships with the community of young people. They undertook a series of initial trauma training sessions with the Consultant Clinical Psychologist, focused on specific environmental aspects such as the need for calming spaces, the requirements for privacy for disclosure, and the importance of distraction and diversion (Cole, Eisner, Gregory, & Ristuccia, 2013).

Most critically, the shared principles of TIP and co‐design were highlighted, especially the need to collaborate closely with young people and the need to redress power imbalances between young people and professionals. Through this, the designers gained an understanding of the importance of an empathetic and curious approach with young people in order to understand the nature of this particular community.

The designers also spent several weeks simply attending the youth work sessions to establish relationships and to gain an understanding of the chemistry and power dynamics at play between the young people, the team and the existing space. As a result of the fluidity they witnessed within the youth work sessions, the designers came to quickly understand that any Home Fae Home activities had to be responsive, flexible, and adaptable to maximise engagement. Young people had to be able to choose their level of engagement, their type of input, and the amount of time spent on their contribution.

**Figure 1:**
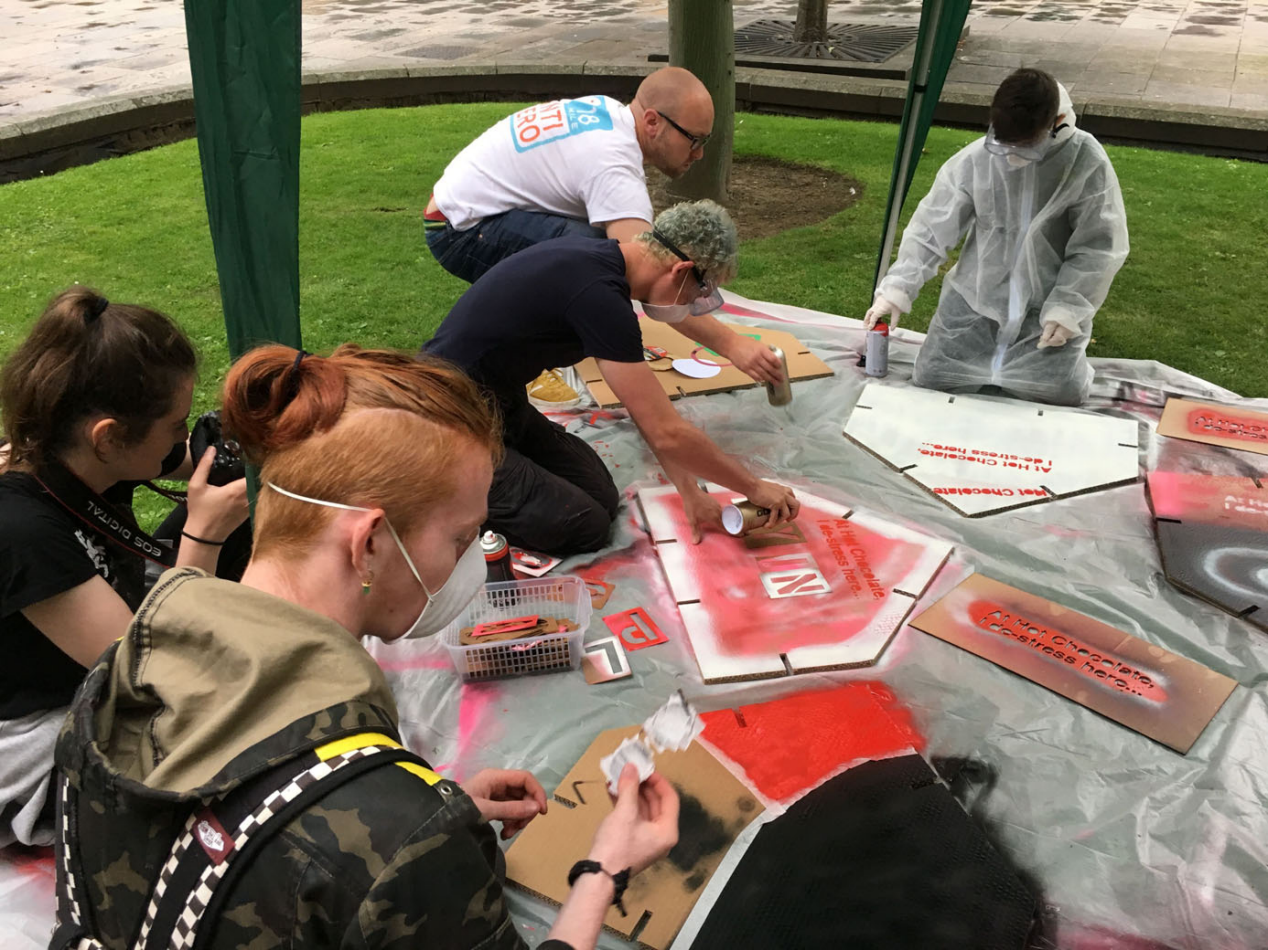
Building Boards (McIntosh, 2019)

**Figure 2:**
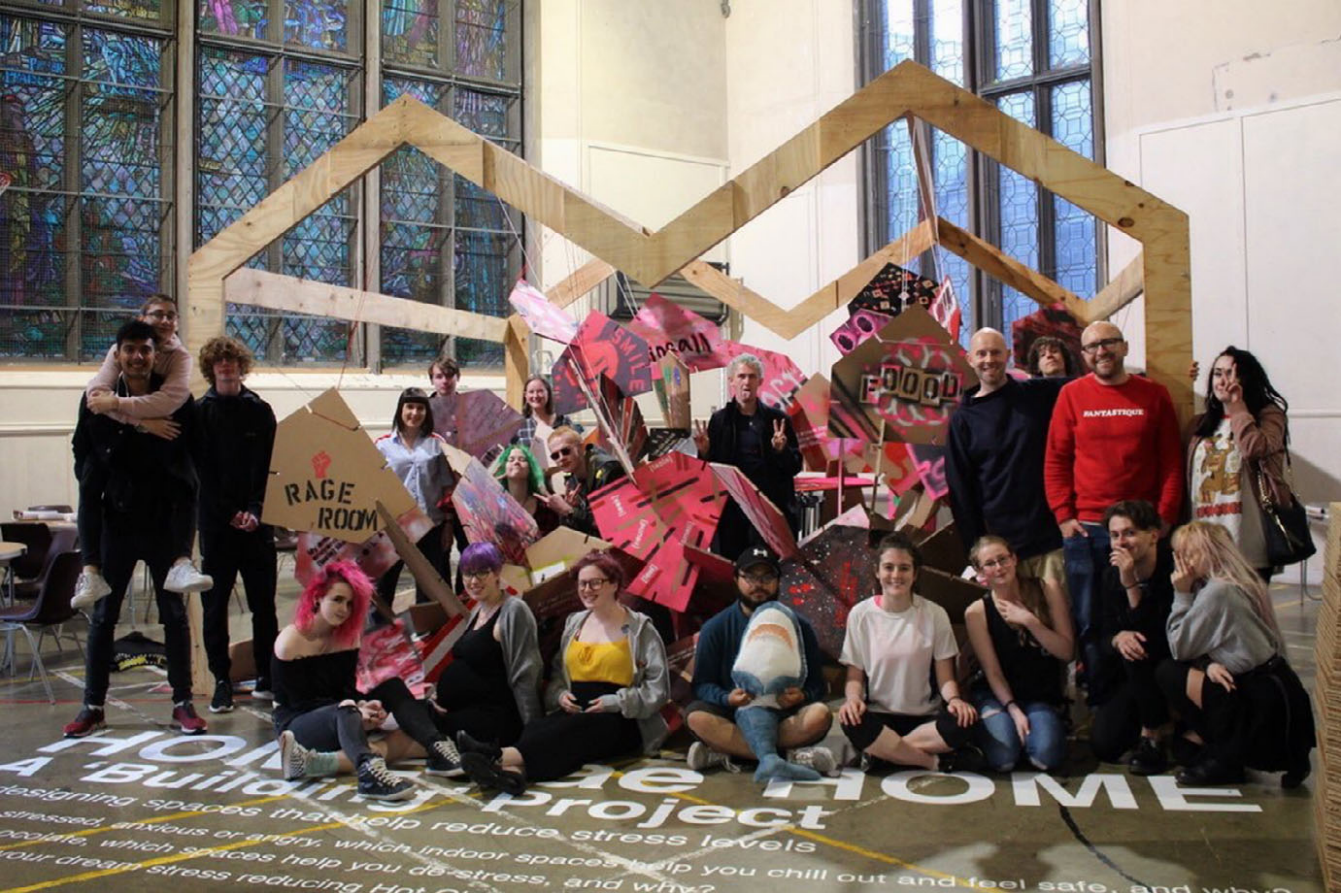
Building Boards Installation (Lyle, 2019)

**Figure 3:**
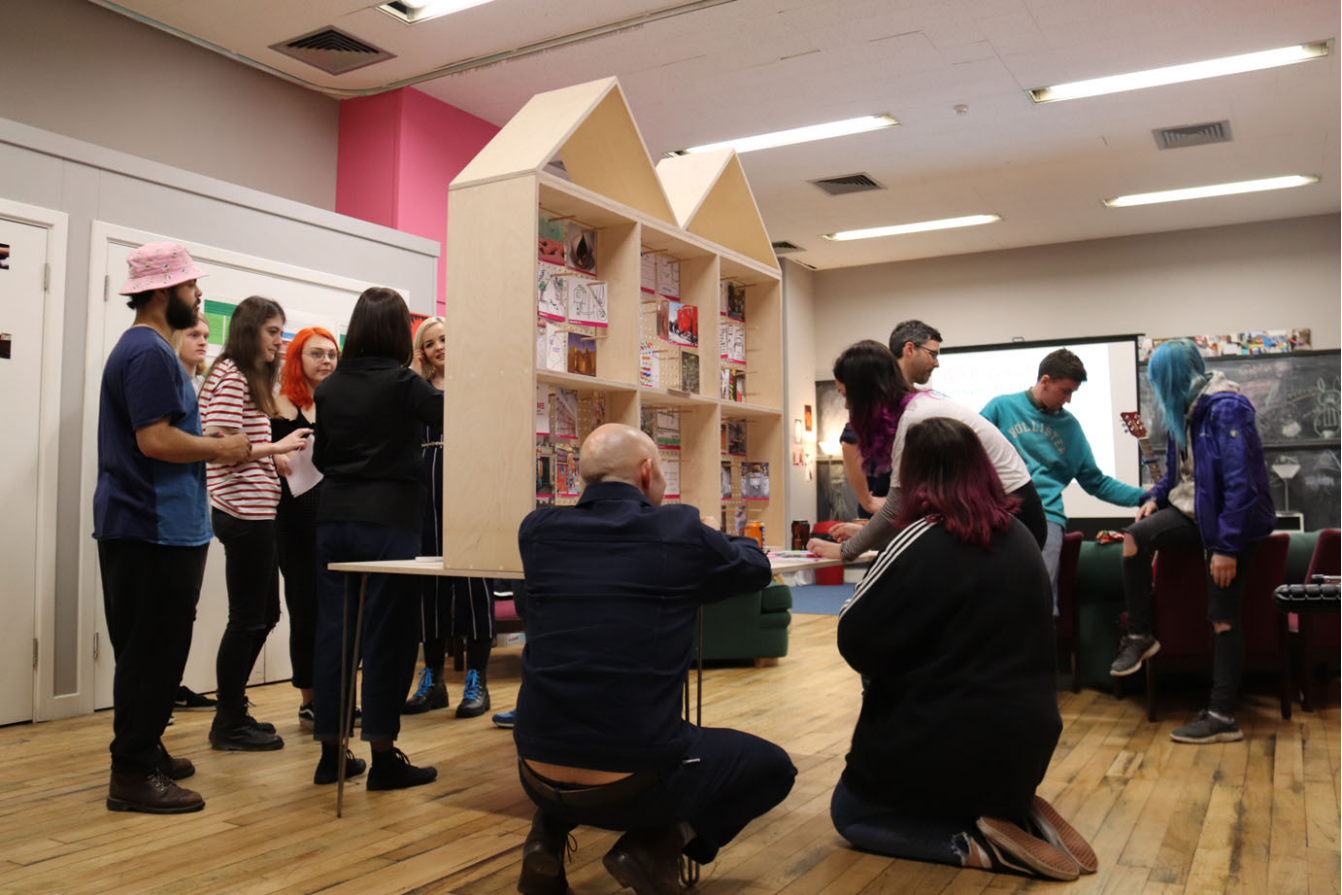
Mood Board (Hackett, 2019)

At the outset of the project, the HCT youth work team was also instrumental in guiding the designers towards a creative ‘language’ that would instantly engage the young people. As a result, spray paint and stencils became the preferred medium of choice for the first round of workshops, where responses to questions around the impact of spaces upon well‐being were expressed on cardboard ‘Building Boards’ (see Figure 1), which slotted together to create a large‐scale installation (see Figure 2). The sense of creating something of size together, within a lengthy project of many years, created the first of a series of ‘quick wins’ for the young people. During a ‘Spatial Safari’ the young people led an exploration of the existing environment to ‘spot’ interesting opportunities, which they documented using Polaroid cameras. They also engaged with the ‘Mood Board’ (see Figure 3), a moveable interactive and immersive station, to consider how key themes generated could look and feel within Hot Chocolate. To express ideas, they could draw, write, or select inspirational imagery, before displaying it on the ‘Mood Board’ for discussion.

Throughout the co‐design process, and after each set of workshops, the designers documented every input from the young people. These were captured mainly by visual methods such as photography, video, and annotated hand‐drawn responses triggered by a spatial image library. Nothing was deemed insignificant, and nothing was discarded. They were organised and analysed, through a process of affinity mapping as laid out by Curedale (2013, pp. 34–35), and then presented back to the community for discussion. This process of reporting back ensured that the young people knew their voices were being heard and that genuine collaboration was maintained.

### Action research

Alongside the co‐design workshops which focused largely on identifying and solving the tangible problems of the space, HCT youth workers also engaged in targeted research conversations with young people to generate new knowledge from their experiences and perspectives about trauma and physical environments. A multi‐methods approach was used, with semi‐structured interviews and focus groups, as well as more creative and informal engagements. Because of the long‐established trusting relationships between young people and youth workers, the data generated was extremely honest, rich, and profound. These conversations were transcribed, and thematic coding analysis was used in order to identify the key themes from the results (Boyatzis, 1998).

## Results

As indicated in the methods section, action research seeks to both generate new knowledge and tangibly solve a problem. The following sections present the outcomes of the ‘problem to be solved’ (the space) and the generation of new knowledge (the young people).

### Space

**Figure 4:**
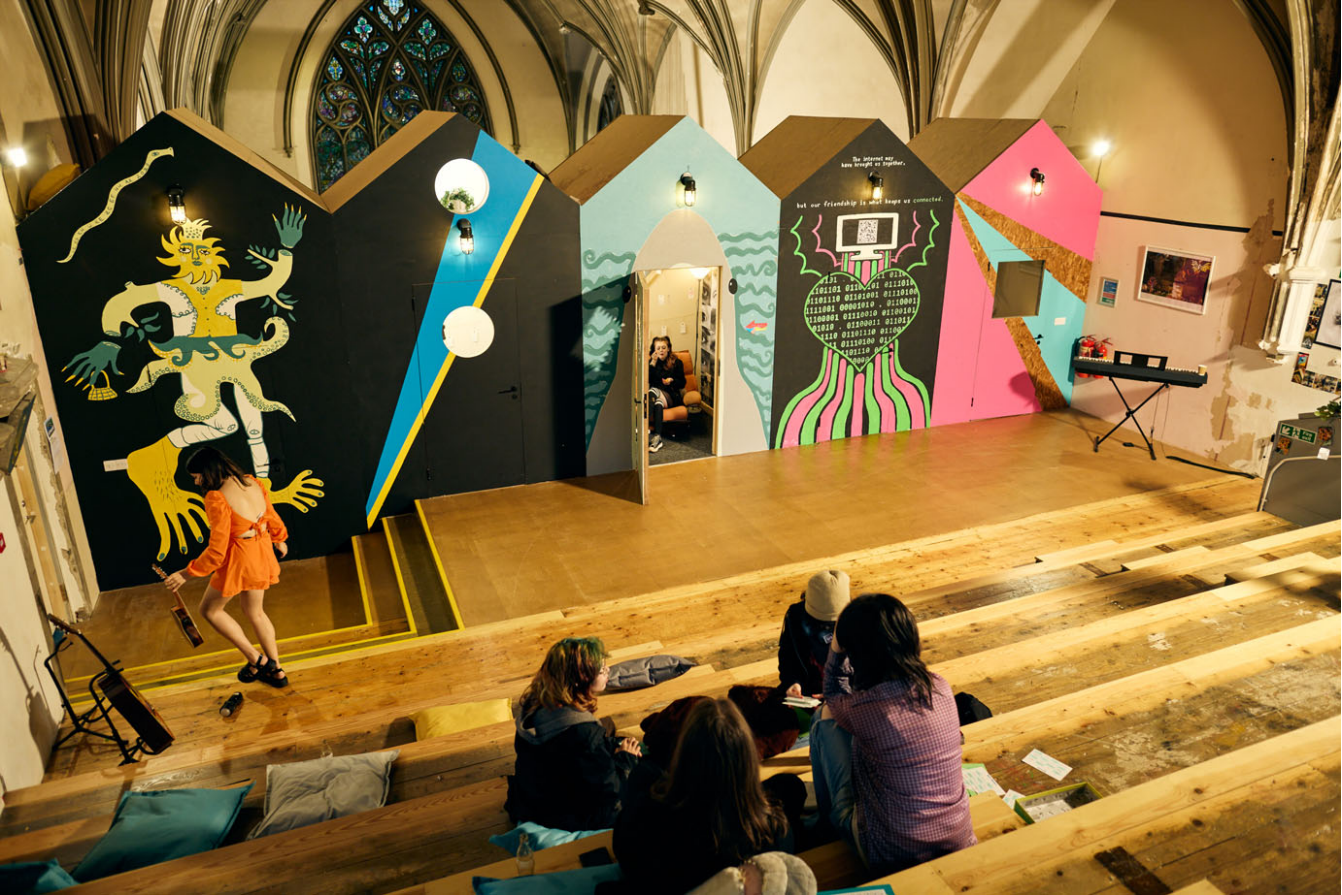
The Steps (Dibdin, 2024a, 2024b)

**Figure 5:**
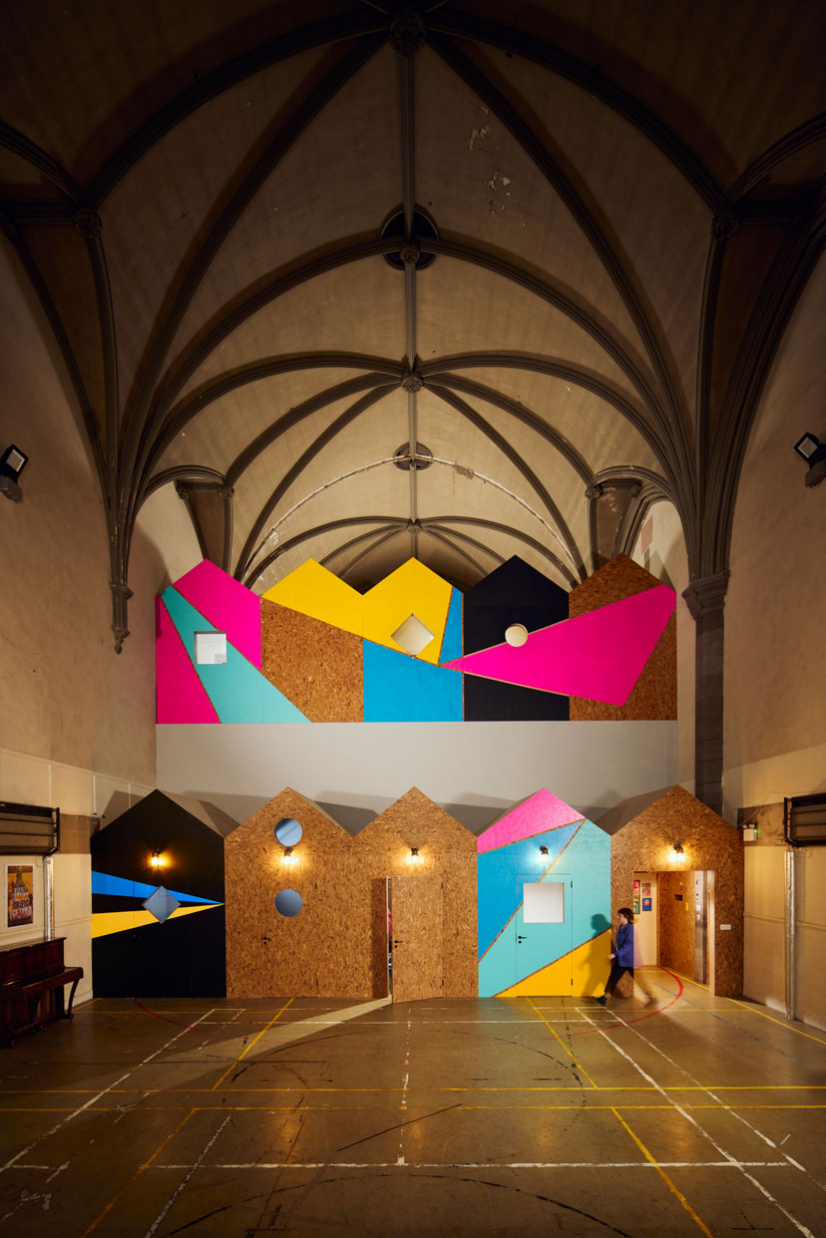
Beach Huts (Dibdin, 2023)

**Figure 6:**
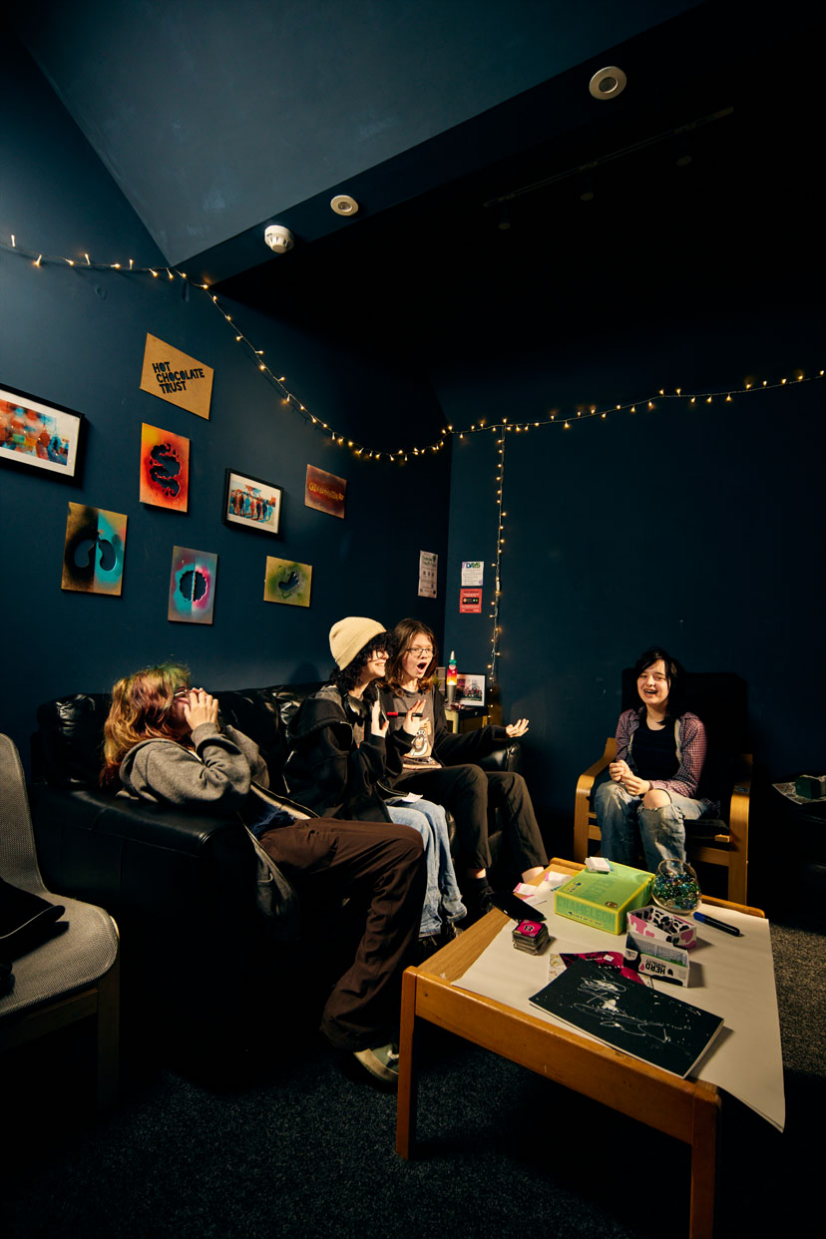
Cocoon Room (Dibdin, 2024a, 2024b)

The space has been utterly transformed, both aesthetically and functionally. The project demolished walls, revealed hidden spaces and stained glass windows, built new structures, and created multiple new/additional diverse and adaptable youth work spaces. Each space feels very different due to the range of sizes, lighting, privacy and function (see Figures 4, 5, 6).

The spaces are precious in terms of what they provide as an experience but are not so precious in that they cannot be personalised by the young people – the space evolves through their mark‐making and interactions with them over time. This ‘low‐fidelity’ approach embraced an honesty to materials that reflects the cultural integrity of HCT, where it is vital that the young people feel a personal connection to their adaptable space and ‘at home’ within it.

### Young people

New knowledge was generated with the young people through reflecting on both the co‐design process and the final outcome of the redesigned space. Distinct themes emerged, many of which correlate closely with the trauma‐informed principles, as outlined in Table 1.

**Table 1:** Data themes

Data theme	Co‐design process	Spatial outcome
Safety	“We're influencing it so we can feel as comfy as we can. That makes us feel safer and more understood”	“Having spaces within spaces makes it feel so much safer. It's like you've got layers”
Trust	[Young person on seeing the architectural drawings for the first time:] “I feel heard. And proud. They've actually listened to us. We've not done a whole project and then they've just gone and done their own thing anyway. They've put what we wanted into the drawing”	“I love how we were the ones that came up with the ideas, and that they actually happened. Not many places take young people as seriously as here”
Empowerment	“It feels good to be part of it. We're basically helping build the future of this place”	“By us making these decisions, it's really about making a place that's for and by us”
Choice	“Not everyone's the same, so it's important that we create different rooms for people that like different things”	“The variety is great – I like how flexible and unspecific it is, that we can make it whatever we want, and keep reinventing it. It's brilliant”
Collaboration	“It's important that we get asked about plans cause this place is for us – team get us involved rather than making decisions and expecting us to like it. The plans look cool as f*ck so far. I think it's gonna be a really good place to have”	“The whole thing looks like it's been built by us and for us”
Additional data themes		
Culture	“Teenagers do things in a very different way. What an adult might perceive as really nice and decorative and really practical, a teenager might not like at all” “We're emos and goths here. It's not meant to be all shiny and pretty”	
Expression	“This place works because it feels like a teenager's bedroom. It's exactly how we like it. We've picked the colours, we've got our photos all over the place. Things move around a lot, but the essence remains the same. You rearrange a bedroom god knows how many times as a teenager. And here you get to do that too”	
Community	“It's not just a building in the centre of town, it's a community. And the community is what makes it what it is. You're stronger together, no matter what sh*t gets thrown at you”	
Legacy	“It'll be great to see the new ones enjoying it in the future too. It's like our legacy. Something we can be really proud of. We want HCT to last forever, for the younger generations to feel safe and feel at home”	

### Wider impact

There have been additional benefits of the project for the practitioners involved. HCT's youth work team have a far greater understanding of the potential usefulness of the physical environment when working with young people. As a result, there is a much more thoughtful and deliberate approach to using space, in keeping with the key tenet from educationalist, John Dewey: “Whether we permit chance environments to do the work, or whether we design environments for purpose makes a great difference.” (Dewey, 1916, p. 19).

## Discussion

This project has generated rich and important data, with multiple layers of potential analysis, due to the multi‐disciplinary makeup of the partnership. It is therefore valuable, when analysing the results, to consider these through the different professional lenses.

### Perspective 1: Youth & community work (Charis Robertson, Hot Chocolate Trust)

From a youth and community work perspective, the most important learning from Home Fae Home has been the analysis of power, co‐design, and social justice.

#### Power

The language of ‘community engagement’ ‘co‐design’ and ‘community empowerment’ is rife in Scotland. However, it is rare for the reality of these experiences by communities to match the rhetoric: professionals and systems struggle to relinquish power and to trust communities.

When considering the specific needs of adolescents, for example, services and spaces tend to be designed by adults. What young people want and need are often very different. Youth spaces and services need to feel more ‘like a teenagers bedroom’, full of expression and meaning and continual reinvention. Adults need to step aside and trust young people.

Culturally, services and spaces tend to be designed by middle‐class professionals. Trauma‐informed spaces need to be culturally contextual. Pastel colours, skylights and motivational posters will not help to ground dysregulated goths, emos and moshers.

One size does not fit all. Each young person will have their own way of processing and reducing their stress levels. A variety of spaces is needed: small and cosy; big and spacious; quiet and reflective; loud and active; solo; sociable. And crucially, the young people will benefit most from having the power to choose what space they need at any given time.

#### Co‐design

Co‐design processes tend to be most beneficial to the people directly involved in the co‐design process. Those that follow after may benefit from the resulting outcomes, but will not experience the same degree of impact or ownership. Home Fae Home has crystallised the tenet that to ensure ongoing ownership, co‐design must be ongoing.

Again, drawing on the metaphor of the teenager's bedroom, young people's spaces need to be reimagined, rearranged and redesigned by each new generation in order to nurture and embed ownership. A simple development of this, resulting from the Home Fae Home project, is for young people to repaint the fronts of the beach huts every 6–12 months using their own designs in a new project called ‘Jabba those huts’.

#### Social justice

Trauma‐informed practice is a highly contested subject in the fields of youth, community, and social work. Much has been written in critique, fuelled by concerns about reductionist approaches, a lack of robust evidence, and an overemphasis on individualized responses without acknowledging the oppressive and unjust wider systems and structures. There is a frustration that the principles newly claimed by trauma‐informed practice (safety, trust, empowerment, choice, collaboration) are not unique to psychology but have, in fact, underpinned many professional approaches for decades (including youth work). And there is also a strong sense of ennui about this being the latest government policy fad, which has created a booming unregulated market of trauma‐informed ‘experts’ selling their services (Bath, 2017; Smith & Monteux, 2023; Tseris, 2019).

For years, HCT has shared many of these concerns and critiques, grappling with their implications for a community where experiences of adversity and trauma are so prevalent. But rather than discounting TIP because of its apparent shortcomings, HCT has found ways to work with and integrate the valuable and effective aspects of TIP within the context of an activist community deeply concerned with social justice. The prevalence of trauma and the accompanying industry is just as much a social justice issue as it is a psychological one. It therefore requires a social justice response, as well as a psychological one. The impact of recognizing injustice collectively, validating justifiable feelings of anger, and seeking to channel these into meaningful activism must not be underestimated in the debate around TIP. The Home Fae Home project is a discrete but important example of this.

### Perspective 2: Architecture & Design (Gary Kennedy & Linsey McIntosh, Designers)

Key learning from an architecture and spatial design perspective has been around the importance of determination, the ‘pancake approach’ and the role of the extraordinary.

#### Determination

The ways in which ‘co‐design’ projects are conducted can vary drastically. It can be catastrophic if the process is undermined by those in authority overriding the collective decisions of the team, resulting in members feeling undervalued and hugely wary of investing in such a process in the future. In this case, the designers' determination and investment grew as they came to understand the responsibility to deliver a project that would reflect the thoughtful and imaginative creative input from the young people, especially in light of the fact they have been so often let down by professionals. This sense of connection and trust, through the strong relationships developed, highlighted the positive impact that design engagement can have on peoples' lives.

In order to achieve a positive outcome, the designers pursued a sensitive and thoughtful collaborative approach with all stakeholders (young people, HCT team and trustees, church leaders as the owners/custodians of the building, funders and contractors), ensuring there was an open and transparent dialogue throughout, with the young people's voices at the heart. As a result, there was a deep commitment from all stakeholders to honour the vision of the young people. This dogged determination was very much put to the test when faced with several serious challenges that could potentially completely derail the project, such as Covid delays, lengthy council permissions, budget pressures, and contractors who went into administration.

The young people were not shielded from these challenges across the project. Instead, they were seen as integral problem solvers, and as such, were also to build their sense of collective determination and resilience.

#### ‘Pancake approach’

The very first co‐design workshop was hosted in the main hall during a drop‐in session whilst other activities were also on offer around the building. During delivery, which was quite structured, the young people quickly dispersed at hearing of the opportunity to make pancakes in the kitchen. The workshop seemed doomed, but in‐the‐moment reflections with the youth workers led to the reimagining, relocating, and reframing of what we were trying to do. This led to enhanced engagement. Reflecting afterwards with the youth workers, it was clear that future workshops needed to be approached in a more flexible manner and that reflecting in and on the moment, experimentation, and adaptation would become an important part of the project's evolution (Brookfield, 2017; Schon, 1991).

#### The extraordinary

The designers' approach to co‐design was what they call ‘proptastic’, whereby they developed a bespoke set of tools, often three‐dimensional and large in scale, which allowed the co‐design team to creatively investigate and collate information. These were specially designed and fabricated to encourage young people to share creative ideas through an extraordinary experience of fun, hands‐on engagement. The approach was based on the belief that everyone has the ability to be part of a design process if provided with the appropriate tools. Careful consideration was given to their development to ensure the right tools for the right people in the right context to ensure the best result. These ‘props’ were used as icebreakers and creative stimulants and to allow the young people to communicate potentially complex feelings towards sensitive subjects.

### Perspective 3: Psychology (Anne McKechnie, Consultant Forensic and Clinical Psychologist)

#### Pioneering

“Start where you are. Use what you have. Do what you can.” This quote, attributed to Arthur Ashe, the first Black tennis player to win a grand slam, resonated with the project team in terms of the pioneering nature of the Home Fae Home project. The dearth of literature on trauma‐informed environments when we began the project meant there was a conundrum from the outset: How to develop something evidence‐based, when there is almost no evidence? And even now, the latest policy document published by NES (2023) *Roadmap for Creating Trauma‐Informed and Responsive Change* says very little about the physical environment.

Ashe's words offered courage that we could (and must) have confidence in our expertise. We must draw on past professional experiences, learn from them, and translate them creatively into the Home Fae Home project.

I consult to the Scottish Child Abuse Inquiry, a legal entity launched in 2015 that uses a trauma‐informed approach to taking and interpreting evidence from adults who were abused as children in care as well as evidence from other witnesses and alleged/convicted offenders. The Inquiry's main facility was designed to have open, airy, well‐signed places with neutral colours, natural planting and artwork produced by the children of staff members. A young person's secure unit, where I also worked for years, had yet another design brief for its context. Both these physical environments served those contexts well.

But the community of HCT has a very different purpose, culture, and way of working. One of my advisory tasks for the Home Fae Home project was, therefore, how to use the useful learning from the Inquiry, secure unit, and other settings in which I had gained clinical experience, whilst also meeting the needs of a dynamic and fluid community of young people with differing requirements and expectations.

#### Redressing power imbalances

Connecting with the experiences and ideas of the young people was central to ‘translating’ the learning from other contexts, in accordance with the TIP principles of choice, collaboration, and empowerment (NES, 2017). As an integral part of the project, I spent time in informal conversation with the young people to gauge views on the process as well as the actual design and build. Knowing that many young people who have faced trauma and adversity could be wary of talking to a clinician due to negative past experiences and power imbalances, I discarded my official job title and assumed the role of Home Fae Home team member. My conversations were deliberately focused on their views of the proposed renovations with no mental health content or intent. Through deliberately redressing this power imbalance, and by validating the young people's experiences as central to the process, I and the project team gained a depth of insight into their hopes and needs far beyond those that a ‘normal’ clinician may have.

#### Mutlidiciplinary approaches

My previous experience in working with trauma‐informed contexts has been largely with those who deliver services through health, care or legal avenues. I had worked with HCT for a number of years and was confident in their ability to embed TIP principles into their youth work. However, this was the first time I had worked with creative professionals. It was a challenge to me not only in the ability to translate multi‐disciplinary functioning to areas outside health and social care, but also reinforced the goal of the trauma training framework in Scotland that “trauma is everybody's business” (NES, 2017). It also spoke to me of the importance of applying the principles of trust, choice, collaboration and empowerment beyond the client group and into working with other agencies and professionals. The project's success was in no small part down to the project team taking time to build trusting relationships with each other, and by respecting each other's unique professional contributions.

#### Leadership

Finally, if we are serious about creating trauma‐informed organisations, it is essential to have buy‐in at all levels. Without senior management recognising the importance of TIP, which is then reflected in organisational policy, practice, and budget, sustained trauma‐informed change will be impossible. This is clearly stated as a requirement in the NES Roadmap (2023).

The Home Fae Home project was an excellent example of the vision and determination demonstrated by HCT's senior leadership team, board of trustees, and youth workers to make TIP a strategic organizational reality.

## Conclusions

The Home Fae Home project has offered revelatory insights directly from young people about the importance and impact of the physical environment when processing and recovering from traumas they may have experienced.

If the key principles of TIP are safety, trust, empowerment, choice, and collaboration (NES, 2017), then trauma‐informed professionals and services from all fields must be the first to embody these.

The learning from this paper can be transferred to many other contexts, including statutory services such as schools and child and adolescent mental health services; community centres; and charities/NGOs. Professionals, services, and community groups must therefore work deliberately to address power imbalances and cultural differences, trusting young people to be the experts of their own experiences as core respected partners of co‐design. Without addressing these power imbalances and prioritising an authentically co‐produced process and spatial outcome, a truly trauma‐informed organisational approach will neither be achievable nor sustainable.

## Funding information

The Home Fae Home project was funded by Life Changes Trust, with additional financial support from Northwood Charitable Trust, The Baird Trust, Alexander Moncur Trust, STV Children's Appeal, Benefact Trust, the Kiltwalk, and the Stephen Fry Public Engagement Awards.

## Conflict of interest statement

The authors have no competing or potential conflicts of interest.

## Ethics statement

The project plan, research design, informed consent processes, data collection, storage, and analysis were all in line with the Community Learning & Development Standards Council of Scotland's Code of Ethics as well as Hot Chocolate Trust's consent and data protection policies and procedures.

## Data Availability

Data sharing not applicable (this features a case study, not a clinical or controlled trial). There is a comprehensive resource bank detailing Home Fae Home's co‐design processes, tools, and techniques, as well as video reflections from the designers and young people available at: http://www.hotchocolate.org.uk/home‐fae‐home.
